# Supplementary material for: Complications of Tumor Nephrectomy with and Without Tumor Thrombus in the Vena Cava, Recorded with the Clavien–Dindo Classification: A Matched-Pair Analysis
Source: Cancers (Basel). 2024 Oct 18;16(20):3523. doi: 10.3390/cancers16203523 (PMC11506320; doi:10.3390/cancers16203523)
Supplement: Supplementary file 1 [file cancers-16-03523-s001.zip › cancers-3214517-supplementary.pdf]

Supplementary Materials

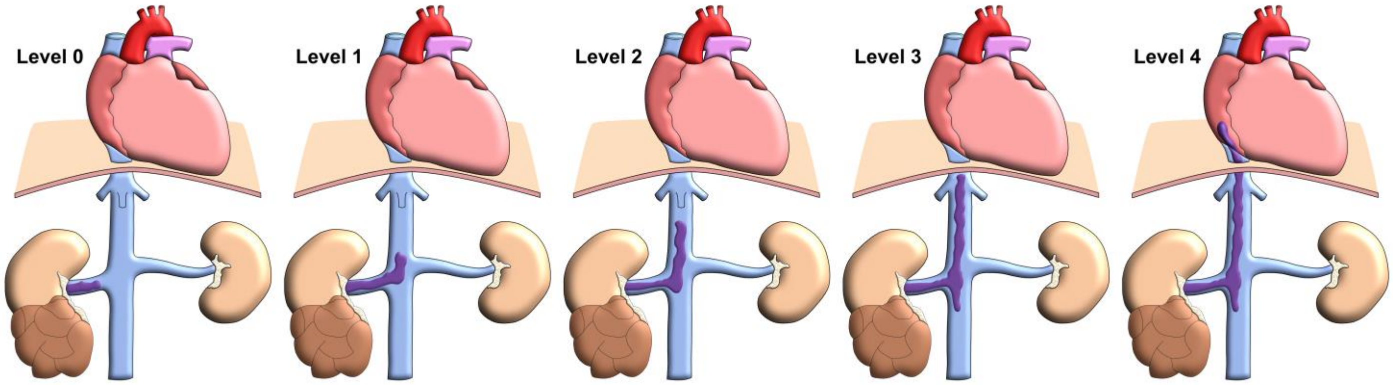

Figure S1. Mayo-classification for tumor thrombus (TT). Level 0, TT in the renal vein; Level 1, TT in the inferior vena cava (IVC) up to ≤2cm cranial of the renal vein; Level 2, TT to >2cm cranial of the renal vein, but not to the hepatic vein; Level 3, TT to cranial of the hepatic vein, but not to the diaphragm; Level 4, TT in the supradiaphragmatic IVC or into the right atrium.

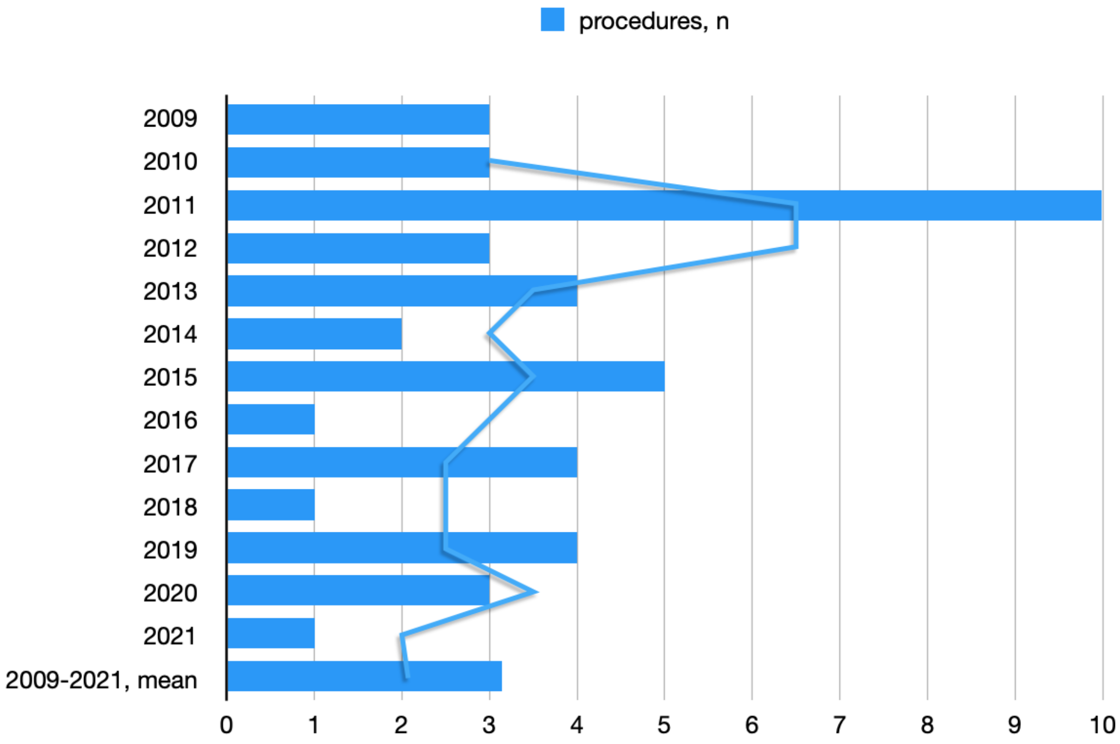

Figure S2. Radical nephrectomies with inferior vena cava thrombectomy per year at Jena University Hospital.

Table S1. Electrolyte imbalances.

	TT-group	Non-TT-group	Overall	p value
	(n = 44)	(n = 44)	(n = 88)	(TT vs. non-TT)
Electrolyte imbalances, n (%)	25 (57)	36 (82)	61 (69)	0.011
CDC-Grade 1 electrolytes, n (%)	25 (57)	36 (82)	61 (69)	0.011

Table S2. Adherence to quality criteria for accurate and comprehensive reporting of surgical outcome [1].

Recommendation	Result
Define the method of accruing data.	Done (see 2.1.).
Define who collected the data.	Done (see 2.1.).
Indicate the duration of follow-up.	Done (see 2.4.).
Include outpatient information.	Rejected with reason: Earlier assessment of postoperative complications demonstrated missing data for later complications with a potential bias due to underreporting, e.g. in the reports of centers of rehabilitation, most of our patients went to after leaving hospital.
Include mortality data and cause of death.	Done (see 3.3 – no patient died).
Include definitions of complications.	Done (see 2.4. and 2.5).
Define procedure-specific complications.	Done (see 2.4, 2.5, 3.3).
Use a severity grading system (avoiding the distinction minor/major); the Clavien-Dindo system is recommended.	Done (see 2.5.).
Postoperative complications should be presented in a table either by grade or by complication type (specific grades should always be provided; grouping is not accepted)	Done (see Fehler: Referenz nicht gefunden)
Include risk factors: American Association of Anesthesiologists (ASA) score, Charlson score, Eastern Cooperative Oncology Group, other.	Done (see 2.2.).
Include readmissions and causes	Not applicable (focus on postoperative complications through to discharge or postoperative day 30, whatever came first)
Include reoperations, types and causes	
Include the percentage of patients lost to follow-up	

References

1. Mitropoulos, D.; Artibani, W.; Graefen, M.; Remzi, M.; Roupret, M.; Truss, M.; European Association of Urology Guidelines Panel. Reporting and grading of complications after urologic surgical procedures: An ad hoc EAU guidelines panel assessment and recommendations. *Eur. Urol.* **2012**, *61*, 341–349. https://doi.org/10.1016/j.eururo.2011.10.033.
